# Morphological parameters and anatomical locations associated with rupture status of small intracranial aneurysms

**DOI:** 10.1038/s41598-018-24732-1

**Published:** 2018-04-24

**Authors:** Zhihui Duan, Yuanhui Li, Sheng Guan, Congmin Ma, Yuezhen Han, Xiangyang Ren, Liping Wei, Wenbo Li, Jiyu Lou, Zhiyuan Yang

**Affiliations:** 1 Department of Neurology, Affiliated Luoyang Central Hospital of Zhengzhou University, Luoyang, Henan P.R. China; 2 Department of Neurology, The 2nd Affiliated Hospital of Zhengzhou University, Zhengzhou, Henan P.R. China; 3 Department of Interventional Neuroradiology, The 1st Affiliated Hospital of Zhengzhou University, Zhengzhou, Henan P.R. China; 4 Department of Interventional Neuroradiology, Affiliated Luoyang Central Hospital of Zhengzhou University, Luoyang, Henan P.R. China

## Abstract

Characterization of the rupture risk factors for small intracranial aneurysms (SIAs, ≤5 mm) is clinically valuable. The present study aims to identify image-based morphological parameters and anatomical locations associated with the rupture status of SIAs. Two hundred and sixty-three patients with single SIAs (128 ruptured, 135 unruptured) were included, and six morphological parameters, including size, aspect ratio (AR), size ratio (SR), height–width ratio (H/W), flow angle (FA) and aneurysm width–parent artery diameter ratio, and the aneurysm locations were evaluated using three-dimensional geometry, and were used to identify a correlation with aneurysm rupture. Statistically significant differences were observed between ruptured and unruptured groups for AR, SR, H/W, FA, and aneurysm locations, from univariate analyses. Logistic regression analysis further revealed that AR (*p* = 0.034), SR (*p* = 0.004), H/W (*p* = 0.003), and FA (*p* < 0.001) had the strongest independent correlation with ruptured SIAs after adjustment for age, gender and other clinical risk factors. A future study on a larger SIA cohort need to establish to what extent the AR, SR, H/W and FA increase the risk of rupture in patients with unruptured SIAs in terms of absolute risks.

## Introduction

Intracranial aneurysm (IA) is a prevalent vascular disorder affecting 2–5% of the population worldwide^1,2^, and IA rupture frequently leads to fatal subarachnoid hemorrhage (SAH). With the widespread use of advanced imaging scanning, unruptured IAs are incidentally discovered with an increasing frequency. In a 5,720 cohort study, 91% of the IAs were identified incidentally, and close to 50% of them are <5 mm in diameter^3^, which are defined as small IAs by published reporting standards^4,5^. Results from the large cohort studies, including those of the International Study of Unruptured Intracranial Aneurysms (ISUIA), indicate that aneurysms smaller than 5 or 7 mm rarely rupture and thus surgical and endovascular treatments are rarely justified^3,6,7^. However, discrepant data shown that small IAs account for 13% of all ruptured IAs by two independent studies long time ago^8,9^, and more recent studies also reported a higher percentage of small IAs among their ruptured cases^10–13^. In addition, most of the ruptured IAs seen in daily clinical practice are small IAs^10,14^, and small IAs are a common cause of aneurysmal SAH^15^. To determine the most appropriate management plan for individual patients, we need to have a better understanding of the rupture risk of small IAs.

Numerous studies in analyzing the morphological characteristics of IAs have demonstrated that geometric parameters such as aspect ratio (AR)^16,17^, size ratio (SR)^18,19^, and aneurysm inflow angle (FA)^20^ are associated with aneurysm rupture status. However, most of these previous studies were not focused on evaluating the rupture-associated morphological parameters specific to small IAs (<5 mm). Additionally, reported results on the aneurysm locations associated with rupture status are conflicting for small IAs. Although most of the studies agreed that the most prevalent location of ruptured small IAs was anterior communicating artery (AcoA)^21,22^, in a 446 small IAs cohort study, the percentage of ruptured small IAs locating at AcoA was considerably lower than those at internal carotid artery (ICA) and middle cerebral artery (MCA)^23^. Nahed. *et al*., reported in their 100 cohort study that the posterior communicating artery (PcoA) small IAs were 3.5 times more likely to rupture than the AcoA ones^24^. Because location of an aneurysm has been approved to predict both surgical and endovascular outcomes, the conflicting observations on the aneurysm locations associated with rupture status for the small IA group warrants further investigation.

The aim of the present study is to identify image-based morphological parameters and anatomical locations associated with the rupture status of SIAs.

## Results

263 patients with small IAs (<5 mm) were included in the study with 128 ruptured and 135 unruptured aneurysms. Demographic and clinical information of the study population is listed in Table 1. Mean age of the patients was 60.19 ± 7.18 years (range, 41–81years), and 49.8% (131/263) were women. A total of 48.7% (128/263) SIAs were ruptured. None of the clinical risk factors showed significant correlation with the rupture status (*p* > 0.05).

**Table 1:** Demographic information and clinical risk factors for patients with ruptured and unruptured small intracranial aneurysms.

	Unruptured (n = 135)	Ruptured (n = 128)	*P* value
Age (SD)	59.84 (7.26)	60.55 (7.11)	0.429
Female (%)	74 (54.8)	57 (44.5)	0.095
Smoking (%)	29 (23.2)	26 (20.3)	0.816
Hypertension (%)	52 (38.5)	62 (48.4)	0.105
SAH history (%)	6 (4.4)	11 (8.6)	0.171
Family history (%)	6 (4.4)	4 (3.1)	0.813

We used univariate analysis to examine pre-defined morphological parameters individually and compared their values between the ruptured and unruptured groups (Table 2). Ruptured SIAs were associated with larger flow angel (FA) (123.68 *vs*.112.19, *p* < 0.001), higher aspect ratio (AR) and size ratio (SR) (1.61 *vs*. 1.49, *p* = 0.008 and 1.86 *vs*. 1.71, *p* < 0.001), and higher height-width ratio (H/W) and (1.50*vs*. 1.43, *p* = 0.008). Aneurysm size (4.28 mm *vs*.4.21 mm*, p* = 0.191) and aneurysm width–parent artery diameter (W/L) ratio (1.58 *vs*.1.51, *p* = 0.061) did not significantly differ between the ruptured and unruptured groups.

**Table 2:** Univariate analyses for the morphological parameters measured for ruptured and unruptured small intracranial aneurysms.

	Unruptured (n = 135)	Ruptured (n = 128)	*P* value
Size	4.21 ± 0.43	4.28 ± 0.42	0.191
AR	1.49 ± 0.37	1.61 ± 0.39	0.008
SR	1.70 ± 0.33	1.86 ± 0.34	<0.001
FA	112.19 ± 16.523	123.68 ± 17.10	<0.001
H/W ratio	1.43 ± 0.24	1.50 ± 0.18	<0.008
W/L ratio	1.50 ± 0.32	1.58 ± 0.40	0.061

**Figure 1:**
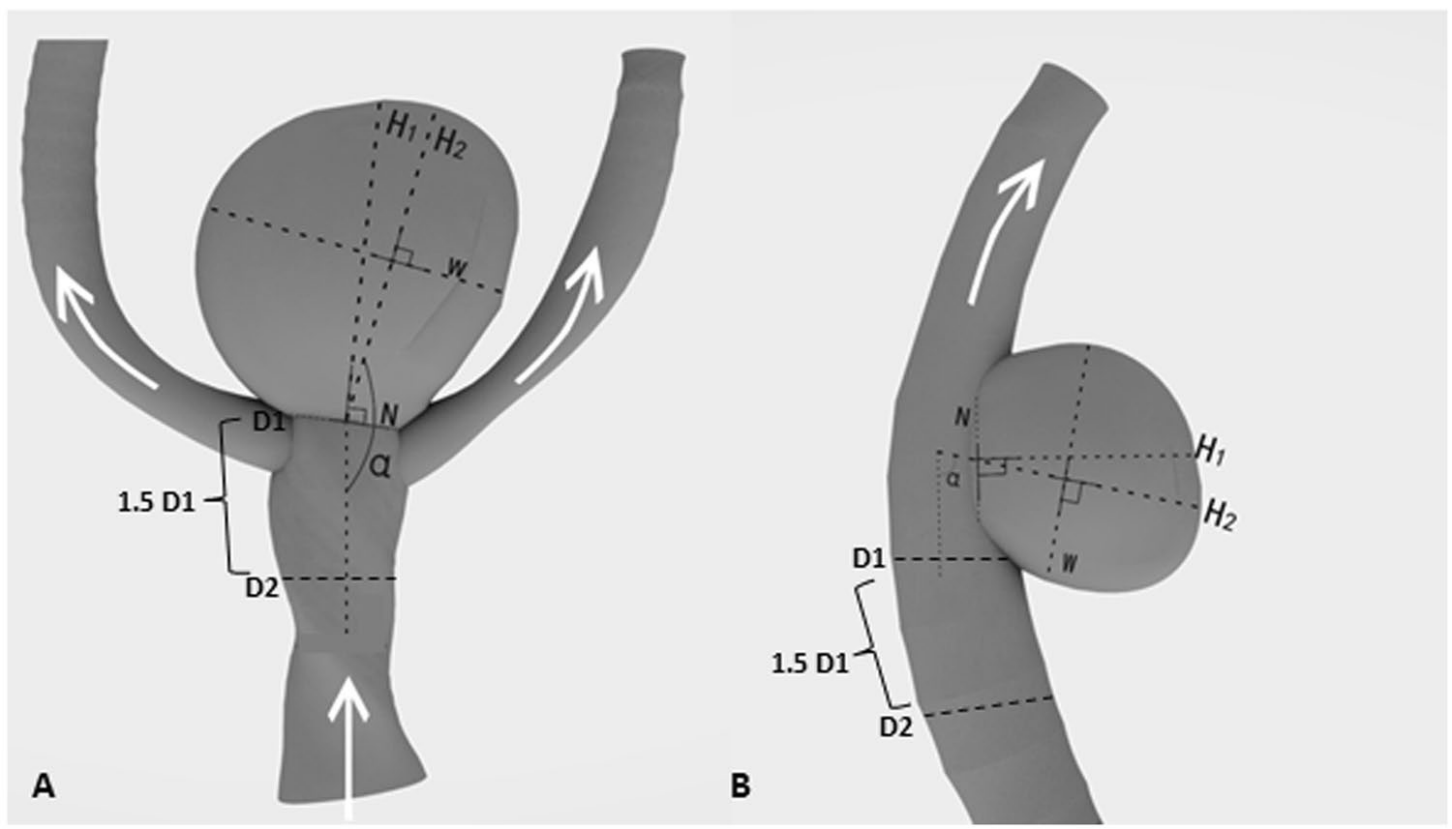
Definition of the morphological parameters in end-wall aneurysm (**A**) and side-wall aneurysm (**B**). Aneurysm size is defined as the maximum distance of the dome from the aneurysm neck plane (W). Aspect ratio (AR) is defined as the maximum perpendicular height (H1) of the aneurysm divided by the average neck diameter of the aneurysm (N). D1: the diameter of the parent vessel at the edge of the neck, perpendicular to flow; D2: the diameter of the parent vessel perpendicular to flow, measured at 1.5D1 from D1; average diameter: (D1 + D2)/2; size ratio (SR) is defined as the maximum aneurysm height (H2) divided by the average diameter. Height–width (H/W) ratio is defined as the ratio of H2/W. Aneurysm width–parent artery diameter ratio is the ratio between W/D1. Flow angle (FA) is defined as the angle between the maximum height of the aneurysm and the parent vessel (α).

In considering that the FA of side-wall aneurysm is different from that of end-wall one (Fig. 1), and the percentages of each type SIAs were different between the ruptured and unruptured groups (71.6% *vs*. 49.3% for side-wall SIAs, and 27.4% *vs*. 50.7% for end-wall SIAs), we further separately analyzed whether the ruptured SIAs are associated with specific type of SIA. The results showed that a larger FA was associated with the end-wall ruptured SIAs (*p* < 0.05).

The χ^2^ test showed a significant relationship between the aneurysm location and aneurysm rupture (χ^2^ = 47.52, *p* < 0.001) (Table 3). Especially, 41.4% (53) and 39.1% (50) of the 128 ruptured SIAs were located at AcoA and PcoA, respectively. PcoA is usually defined as small arteries. However, we recognized that aneurysms at the origin of internal carotid-posterior communicating artery (IC-PC) are internal carotid artery (ICA) aneurysm, and these aneurysms’ SR is not so high because the ICA’s diameter is usually big. In checking our dataset, we confirmed that the SR of aneurysms at PcoA was indeed higher in the ruptured group than that in the unruptured groups (*p* < 0.05).

**Table 3:** Univariate analyses for the locations of the ruptured and unruptured small intracranial aneurysms.

	Unruptured (n = 135)	Ruptured (n = 128)	χ^2^	*P* value
ACoA	16	53	47.526	<0.001
PCoA	56	50		
BA	9	5		
Ophthalmic artery	30	4		
MCA	8	3		
ACA	8	6		
Cavernous carotid artery	4	3		
Others	4	4		

In the multivariate logistic regression model adjusted for both morphological and clinical risk factors, FA, SR, H/W ratio and AR were evaluated as independent variables and the results were summarized in Table 4. The analysis showed that greater SR (OR 3.586; 95% CI 1.518 to 8.474, *p* = 0.004), larger FA (OR 1.045; 95% CI 1.026 to 1.064, *p* < 0.001), higher H/W ratio (OR 8.023; 95% CI 2.011 to 32.008, *p* = 0.003), and AR (OR 2.241; 95%CI 1.065 to 4.715, *p* = 0.034) had the strongest independent correlation with ruptured SIAs.

**Table 4:** Multivariate analysis (Logistic regression) after adjustment for clinical and morphological risk factors.

	OR (95% CI)	*P* value
SR	3.586 (1.518–8.474)	0.004
FA	1.045 (1.026–1.064)	<0.001
H/W ratio	8.023 (2.011–32.008)	0.003
AR	2.241 (1.065–4.715)	0.034
Age	1.023 (0.983–1.065)	0.255
Smoking	1.158 (0.579–2.315)	0.678
Hypertension	1.505 (0.849–2.668)	0.161
SAH history	1.978 (0.591–6.625)	0.269
Family history	0.922 (0.223–3.816)	0.911
W/L ratio	1.100 (0.482–2.509)	0.821
Female	0.625 (0.350–1.118)	0.113
Diameter	1.513 (0.770–2.974)	0.230

## Discussion

In this study, we identified that aneurysm FA, SR, AR and H/W ratio were significantly different between ruptured and unruptured SIAs. Moreover, ruptured SIAs were more likely to locate at AcoA. To our best knowledge, there were only a few reports about morphological parameters of SIAs in evaluating the rupture risk, the results of the current study may imply that, for SIAs that are equally suitable for observation or endovascular treatment, having these morphological risks factors and being located at AcoA site may need closer monitoring or more prompt intervention.

Controversy exists in the management of patients with unruptured SIAs, and one of the reasons is because SIAs were generally considered benign or “safe” with a very low rupture risk (0–0.05% per year)^3,6,7^. However, Pavis *et al*. recently did a cross-sectional hospital-based study of aneurysmal SAH in emphasizing the aneurysm size at the time of rupture, and the results clearly indicate that SIAs are a common cause of aneurysmal SAH^15^. In addition, extensive data have demonstrated that the rupture risk of a IA depends on a number of biological factors in addition to aneurysm size^25–27^, implying that the prevailing notion of “the larger the IAs, the greater the rupture risks”, is not entirely true. From 2009 to 2017, we encountered total 310 ruptured IAs in our hospital, and about 41.3% (128/310) of them are SIAs, also indicating the prevalence of rupture from SIAs in the Chinese demographic setting. One possible explanation for the high rupture rate of SIAs is that aneurysm rupture occurs relatively soon after formation when the aneurysm wall is weaker and before healing processes takes place. After this initial stage, aneurysms may reach a somewhat stable condition. This is ascertained by the observation that the rupture risk of unruptured IA is higher during the first year after diagnosis and decreases thereafter^28,29^.

Our study didn’t identify any significant known demographic and clinical risk factors for SIA rupture, including age, gender, family history, smoking, hypertension and prior history of SAH. While focused studies on SIAs are scarce, our results are concordant with the general conclusions from the 6,413 patients crossing 283 institutions Japanese cohort study^3^. Still, reports on the SIA-associated demographic and clinical risk factors are controversy. For example, Ohashi *et al*.^30^ found that the proportion of ruptured IAs <5 mm was significantly higher among patients with poorly controlled hypertension. Lee, G. J *et al*.^31^ also reported that hypertension was significantly associated with SAH in a very small IAs group. These results, however, diverge from several other studies in which the size of ruptured IAs was reported to have no association between hypertension^32,33^. It’s noted that incidentally discovered SIAs are now frequent in clinic due to the widespread imaging scanning, and such emerging SIA patient population is relative “healthy” lacking hypertension or prior history of SAH. But major presentations of these patients often include overwork without having enough sleep and overstress. The associations between SIA rupture with such social factors may worth further exploration. Overall, the lack of inconsistency in reporting the correlations with known demographic and clinical factors indicates the complex nature of SIAs, and also justifies the seeking for other concomitant biological factors in predicting the rupture.

Among several known morphological parameters, we identified statistically significant independent correlations for AR, SR, H/W ratio and FA with ruptured SIAs. The AR reflects the depth-to-neck ratio, and most of the studies agreed that a higher AR is correlated with a higher risk of rupture^34,35^. A commonly used threshold value is AR = 1.6, above which the risk significantly increases. This relation can be explained that the smaller the neck, the slower the flow, thus, a higher AR seems to reflect a lower intra-aneurysmal blood flow and subsequently a higher risk of rupture^34,35^. In our study, the average AR of ruptured aneurysm is 1.61 ± 0.39; both univariate (*p* = 0.008) and multivariate logistic regression analysis (*p* = 0.034) revealed that AR had a strong independent correlation with ruptured SIAs. The AR offers an advantage over the size that it remains significant regardless of the location, so attentions should be taken for patients with unruptured intracranial aneurysms with AR more than 1.6.

The SR, by including the parent vessel geometry, reflects combined information of aneurysm size and location. For two aneurysms of the same size, a high SR indicates a small-sized parent artery^36^. In our study, logistic regression analysis showed that ruptured SIAs higher SR than the unruptured ones (*p* = 0.004). It’s well recognized that aneurysms arising from smaller vessels have thinner walls, and thus have lower resistance to rupture under a given pressure^37^. This indicates that those aneurysms with a small size of parent artery are smaller and always rupture at a smaller size than those at other locations^38,39^. In our analysis of SIAs’ location, the results showed that 11.9% and 41.4% of the total SIAs located at PcoA and AcoA, respectively, and SIAs at these two locations had high rupture rates (41.4% and 39.1%, respectively). The location and SR data are in accordance with each other, because the PcoA and AcoA are defined as relative small arteries in brain, and previous studies consistently showed that IAs located at the AcoA and PcoA had a high SR and ruptured IAs at these same locations were of smaller size^22,40–44^.

Flow angle is another parameter that incorporates the relation of the aneurysm dome to the parent vessel, for which their spatial relationship has been shown to be an important determinant of flow patterns inside the aneurysm dome^20,45^. Merih *et al*. did a computational fluid dynamics modeling and showed that increasing inflow angle would lead to a deeper migration of the flow recirculation zone into the aneurysm, and result in higher inflow velocity and wall shear stress in both the inflow zone and dome, thus causing rupture^10^. In our study, both univariate and multivariate logistic regression analysis revealed that inflow angle had a strongest independent correlation with ruptured SIAs. Taken together, these results indicate that the relationships with parent vessels, i.e., locating at small parent vessels (PcoA and AcoA) and having a big flow angle, are very critical in determining the rupture behavior of small IAs.

The H/W ratio usually indicates the shape complexity of an aneurysm, which has been reported to be a significant predictor of rupture although not focusing on SIAs^46^. Fusiform aneurysms typically exhibit high H/W ratios whereas lateral aneurysms exhibit low H/W ratios. Regular or lateral aneurysms exhibit a simple flow dynamic of unchanging flow jet direction with a single associated vortex. In contrast, irregular aneurysms or those with a daughter sac have a complex flow dynamic showing changes in the direction of the inflow jet with the generation or destruction of a single vortex or multiple vortices^47^. Thus, aneurysms with high H/W ratios or daughter sacs are likely to rupture^48,49^. In the present study, we didn’t observe any SIAs with obvious daughter sacs, but many of them were irregular shape, and the average H/W ratio of the ruptured SIAs was significant higher than that of the unruptured ones (*p* = 0.003), indicating the potential value of using H/W ratio as a clinical predictor for SIA rupture risk.

Our study has important implications for clinical practice. Small IAs are more and more incidentally discovered in clinical screening, and they are not rare to rupture as underestimated before, but they were undervalued for proper management. Our study focused on identifying imaging-based measurable risk factors associated with rupture of SIAs, thus will provide clinical guidance in further differentiating the high-risk patients and make most appropriate management plan for individual patients. A future study on a larger SIA cohort need to establish to what extent the AR, SR, H/W ratio and FA increase the risk of rupture in patients with unruptured SIAs in terms of absolute risks.

## Materials and Methods

The study was conducted in accordance with relevant guidelines and regulations, and was approved by the Ethics Committee of Affiliated Luoyang Central Hospital of Zhengzhou University. Written informed consent was obtained from each study patient.

### Patient Population

From January 2009 to March 2017, angiography images of patients with single IAs that were diagnosed or treated and present in our database were carefully reviewed. The largest aneurysm size was measured by 2D or 3D angiography, and only patients with aneurysm size ≤5 mm were included in the present study. Such criteria was based on the reporting standards of small IAs in most published studies^4,5^. Subsequently, 263 patients with small IAs were included with 128 ruptured and 135 unruptured aneurysms. Medical records were reviewed to obtain demographic and clinical information, including age, gender, family history, smoking status, hypertension and prior history of SAH. Re-operated aneurysms, fusiform aneurysms, blood blister-like aneurysms, or those associated with arteriovenous malformations were excluded from the study.

### Reconstruction of 3D models

Three-dimensional (3D) models of SIAs were reconstructed from digital subtraction angiography using Siemens Artise Zee software or TOSHIBA AQUILION ONE 320 CTA. Detailed methods of constructing and refining the 3D models have been described previously^50^. All measurements were obtained independently by two observers, and the average value was used for subsequent statistical analyses.

### Definition of parameters and calculation

Six morphological parameters and aneurysm locations were examined in 3D aneurysm models.

These parameters have been defined previously^16–20^ and are described briefly below (Fig. 1). Aneurysm size was defined as the maximum distance of the dome from the aneurysm neck plane. Aspect ratio (AR) was calculated from the maximum perpendicular height of the aneurysm divided by the average neck diameter of the aneurysm. Size ratio (SR) was calculated from the maximum aneurysm height divided by the mean vessel diameter of all branches associated with the aneurysm. The maximum height is the maximum distance from the cross-section of the aneurysm neck to any point on the aneurysm dome. In the case of a terminal aneurysm, the average diameter of the feeding artery and the other branching vessels was used for the ‘average vessel diameter’ in our study. Height–width (H/W) ratio was defined as the ratio of height (the maximum perpendicular distance of the aneurysm dome from the neck plane) to the width of aneurysm, where the aneurysm width was the maximum width parallel to the neck. Aneurysm width–parent artery diameter (W/L) ratio is the ratio between the aneurysm diameter and the associated vessel diameter. Flow angle (FA) was defined as the angle between the maximum height of the aneurysm and the parent vessel. In our study the SIAs were divided into eight location groups: PcoA, AcoA, MCA, ACA, Cavernous carotid artery, ophthalmic artery (OA), basilar artery (BA) and other locations.

### Statistical Analysis

Data are presented as mean and SD for quantitative parameters, and as frequency for categorical parameters. The Kolmogorov–Smirnov test for normal distribution was performed for all quantitative parameters. Student’s *t*-test was used if a parameter was normally distributed; otherwise, a Mann–Whitney *U* test was used to compare differences between ruptured and unruptured lesions. For categorical parameters, the chi-square test was used to analyze the data. Univariate analysis was performed to compare the value of each morphological parameter between the ruptured and unruptured groups. Multivariate logistic regression was used to calculate the odds ratios (ORs) and 95% confidence intervals (95% CI) for the likelihood of aneurysm rupture after adjusting for age, sex, smoking status, family history, hypertension, and prior history of SAH. Results were considered statistically significant at *p* < 0.05. Statistical analysis was carried out using SPSS, Version 17.0 (SPSS, Chicago, IL, USA).
